# Supplementary material for: Adsorption Characteristics and Mechanisms of Fe-Mn Oxide Modified Biochar for Pb(II) in Wastewater
Source: Int J Environ Res Public Health. 2022 Jul 10;19(14):8420. doi: 10.3390/ijerph19148420 (PMC9316531; doi:10.3390/ijerph19148420)

Supplementary Materials

Title: Adsorption characteristics and mechanisms of Fe-Mn oxide modified biochar for Pb(II) in wastewater

Shang-Feng Tang[a], Hang Zhou[a, b*], Wen-Tao Tan[a], Jun-Guo Huang[a], Peng Zeng[a,b], Jiao-Feng Gu[a,b], Bo-Han Liao[a,b]

[a] College of Environment Science and Engineering, Central South University of Forestry and Technology, Changsha 410004, China

[b] Hunan Engineering Laboratory for Control of Rice Quality and Safety, Changsha 410004, China

Supplementary materials captions

Section 1 Screening of Fe/Mn molar ratio

Section 2 Batch adsorption experiments

Section 3 Data processing

Section 4 Related supplementary tables and figures

Table S1 Adsorption kinetic parameters of Pb(II) removal by FM-BC

Table S2 Adsorption isotherm models fitting of Pb(II) by FM-BC

Table S3 Adsorption thermodynamics parameters of Pb(II) by FM-BC

Figure S1 Pb(II) removal rate by FM-BC

Figure S2 Adsorption kinetic plots of Pb(II) adsorbed by FM-BC

Figure S3 Adsorption isotherms and fitting isotherms for adsorption of Pb(II) by FM-BC

Section 1 Screening of Fe/Mn molar ratio

Accurately weighed 5.0-g biochar (300°C) was infiltrated in nine kinds of $Fe(NO_3)_3$ and $KMnO_4$ mix solutions (40 mL; Fe/Mn molar ratio =1/5, 2/5, 3/5 4/5 5/5, 5/4, 5/3, 5/2, and 5/1), respectively. After stirring for 2 h, the mixture was transferred to a 95°C water bath for 22 h, and then was heated at 300°C for 0.5 h. After cooling, the mixture was washed with deionized water several times and dried at 70°C until constant weight to obtain nine kinds of modified-biochar with different Fe/Mn molar ratio. Nine kinds of modified biochar were used for adsorption experiment, screening out the best Fe/Mn modification ratio. The best Fe/Mn molar ratio was determined based on the adsorption capacities of different biochar.

Section 2 Batch adsorption experiments

Effect of pH on adsorption

FM-BC (0.100 g) was added to 200 mg/L Pb(II) solution (50mL) with pH gradients of 2.0, 3.0, 4.0, 5.0, and 6.0, adjusted with 2.0 mol/L HNO_3 and NaOH, and adsorbed at 298 K for 120 min under constant temperature oscillation. The supernatant was filtered through a microporous membrane and stored in a plastic polyethylene bottle for testing. All treatments were repeated three times, as described below.

Effect of additive amount on adsorption effect

FM-BC (0.025, 0.050, 0.100, 0.200, and 0.400 g) was added to 50 mL Pb(II) solution at pH 5.0 and concentration of 200 mg/L. The adsorption times were 5, 10, 15, 30, 60, 120, 240, 480, and 720 min under a constant temperature oscillation at 298 K.

Effect of coexisting ions on adsorption effect

A series of mixed solutions of Pb(II) (200 mg/L) and a single metal element were prepared to construct a Pb-M coexisting system (M represents Ca, Mg, Cu, and Zn). The concentrations of the M ions were set to 10, 25, 50, 100, and 200 mg/L. FM-BC (0.100 g) was added to the coexisting system and adsorbed at 298 K for 120 min under constant temperature oscillation conditions.

Adsorption kinetics

Fifty milliliters of Pb(II) solution with concentrations of 50, 100, 200, and 400 mg/L and pH

5.0, was prepared and 0.100 g FM-BC was added to adsorb at 298 K for 5, 10, 15, 30, 60, 120, 240, 480, and 720 min.

Adsorption isotherm

A series of Pb(II) solutions with concentrations of 25, 50, 100, 150, 200, 300, and 400 mg/L and pH 5.0 were prepared and 0.100 g FM-BC was added to adsorb for 120 min in a constant temperature shaking table at 288, 298, 308, and 318 K, respectively.

Section 3 Data processing

Calculation of adsorption capacity and removal rate

The adsorption performance of FM-BC was based on the adsorption capacity of Pb(II). The adsorption capacity and removal rate at time t are calculated as follows:

$$q_t=(c_0-c_t)V/m \quad (1)$$

$$R\%=(c_0-c_t)/c_0\times 100\% \quad (2)$$

where qt is the adsorption amount at time t (mg/g); c_0 and c_t are the initial concentration of Pb(II) and the concentration at time t (mg/L), respectively; V (L)is the solution volume; and m(g) is the adsorbent mass.

Kinetic analysis

Pseudo-first-order and pseudo-second-order kinetics were used to study the adsorption kinetics of FM-BC on Pb(II) in wastewater:

Pseudo first order dynamics: $\ln(q_e-q_t)=\ln q_e-K_1t$ (3)

Pseudo second-order dynamics: $t/q_t=1/(K_2q_e^2)+t/q_e$ (4)

where q_t is the adsorption amount of Pb(II) by FM-BC at time t (mg/g); q_e is the adsorption amount of Pb(II) in adsorption equilibrium (mg/g); K_1 is the rate constant of pseudo-first-order kinetics, min^{-1}; and K_2 is the rate constant of pseudo-second-order kinetics, $g\cdot(mg\cdot min)^{-1}$.

Isothermal adsorption analysis

The Langmuir and Freundlich equations were used to model the experimental data as follows:

Langmuir equation： $C_e/q_e=1/(K_L \cdot Q_m)+C_e/Q_m$ (5)

Langmuir parameter K_L dimensionless constant separation factor: $R_L=1/(1+K_LC_0)$ (6)

Freundlich equation: $lgq_e=lgK_f+1/n \cdot lgC_e$ (7)

where C_e is the ion concentration in the solution at adsorption equilibrium (mg/L); q_e is the equilibrium adsorption capacity, mg/g; Q_m is the maximum adsorption capacity calculated by the Langmuir model, mg/g; K_L is the Langmuir parameter, L/mg; and C_0 is the initial concentration of the solution. R_L can determine the type of adsorption; if $0<R_L<1$, the adsorption process is favorable; if $R_L \geq 1$, the adsorption process is unfavorable. K_f and $1/n$ are both Freundlich parameters.

Thermodynamic analysis

The thermodynamic parameters of the adsorption process were calculated using Eqs (8) and (9), as follows:

$\ln K_0=\triangle S^{\theta}/R-\triangle H^{\theta}/(RT)$ (8)

$\triangle G^{\theta}=-RT\ln K_0$ (9)

where $\ln K_0$ is the intercept of the linear relationship between $\ln(q_e/c_e)$ and the adsorption amount, R is the gas constant (8.314 J/ (mol · K), and T is the absolute temperature (K).

Section 4 Related supplementary tables and figures

Table S1 Adsorption kinetic parameters of Pb(II) removal by FM-BC

Initial concentration （mg/L）	q_e(exp) （mg/g）	Pseudo-first-order			Pseudo-second-order		
		K_1	q_e(cal) （mg/g）	R^2	K_2	q_e(cal) （mg/g）	R^2
50	25.57	0.0045	0.049	0.762	0.409	33.33	1.000
100	50.47	0.0039	0.033	0.656	0.980	50.51	0.999
200	94.55	0.0071	0.562	0.824	0.102	94.34	0.999
400	165.88	0.0038	47.300	0.975	0.0005	166.67	0.998

Table S2 Adsorption isotherm models fitting of Pb(II) by FM-BC

Temperature	Langmuir model				Freundlich model		
(K)	Q_m (mg/g)	K_L	R_L	R^2	1/n	K_f	R^2
288	135.14	0.133	0.019~0.231	0.999	0.430	21.174	0.803
298	147.06	0.171	0.014~0.189	0.996	0.435	25.722	0.745
308	149.25	0.233	0.011~0.146	0.997	0.396	31.110	0.714
318	151.52	0.333	0.007~0.107	0.999	0.371	36.392	0.747

Table S3 Adsorption thermodynamics parameters of Pb(II) by FM-BC

Temperature(K)	$\ln K_0$	ΔG^{θ}(kJ/mol)	ΔH^{θ}(kJ/mol)	ΔS^{θ}(kJ/(mol·K))
288	3.304	-7.91	32.15	0.14
298	3.536	-8.76		
308	3.997	-10.24		
318	4.566	-12.07		

Figure S1 Pb(II) removal rate by FM-BC

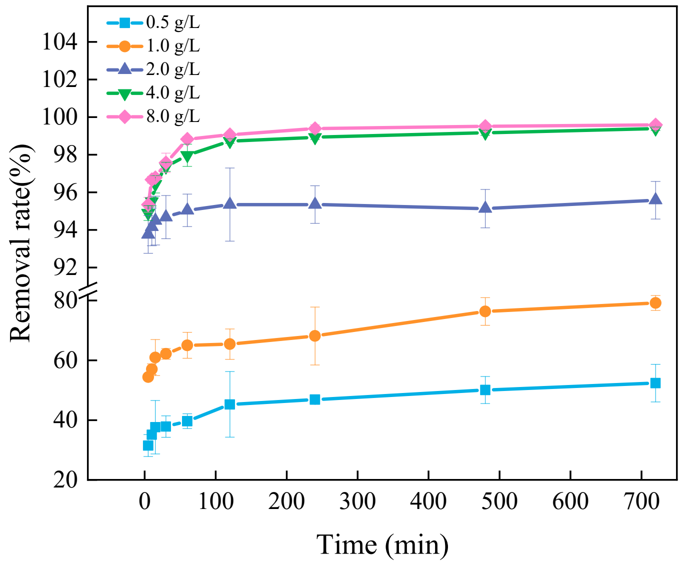

Figure S2 Adsorption kinetic plots of Pb(II) adsorbed by FM-BC

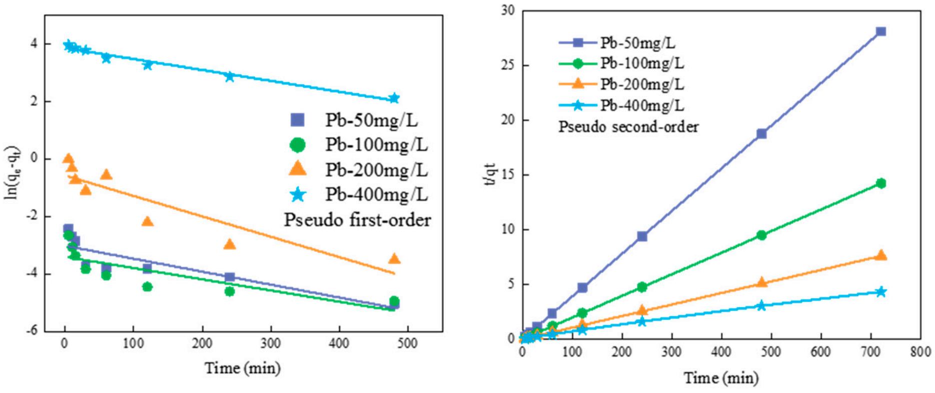

Figure S3 Adsorption isotherms and fitting isotherms for adsorption of Pb(II) by FM-BC

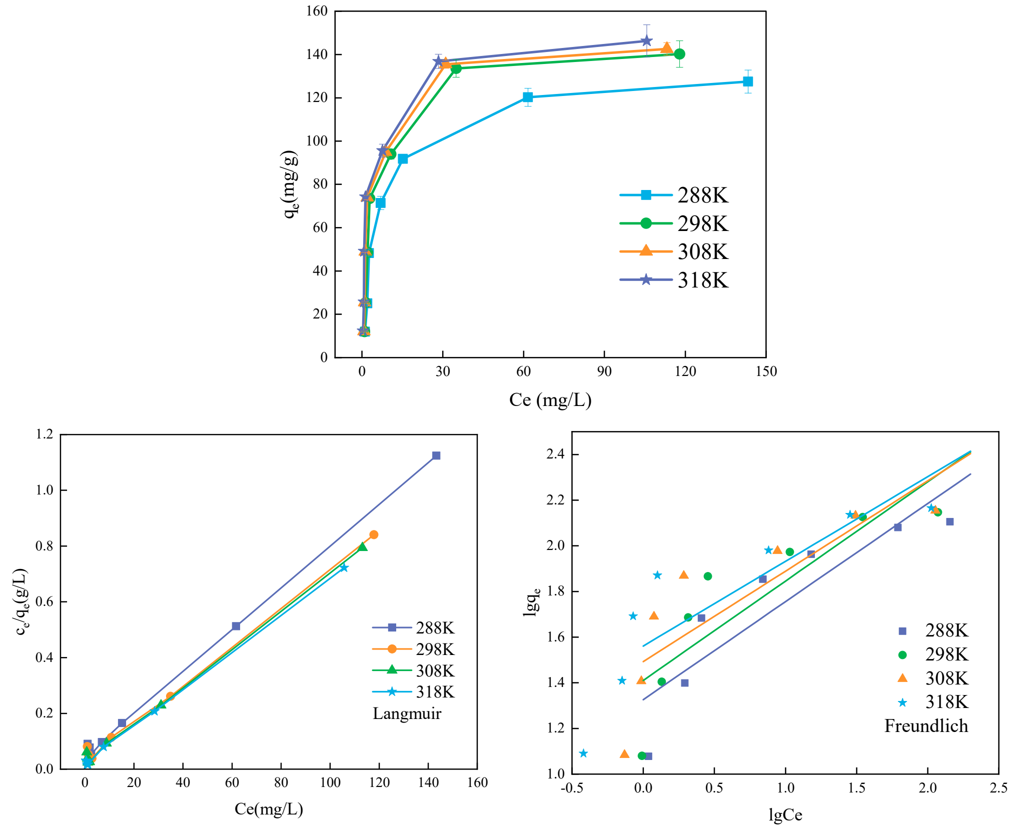

Supplement: Supplementary file 1 [file ijerph-19-08420-s001.zip › ijerph-1764834-supplementary.pdf]
